# Investigating the (cost-) effectiveness of attention bias modification (ABM) for outpatients with major depressive disorder (MDD): a randomized controlled trial protocol

**DOI:** 10.1186/s12888-016-1085-1

**Published:** 2016-11-03

**Authors:** Gina R. A. Ferrari, Eni S. Becker, Filip Smit, Mike Rinck, Jan Spijker

**Affiliations:** 1 Behavioural Science Institute, Radboud University Nijmegen, PO Box 9104, 6500 HE Nijmegen, The Netherlands; 2 Pro Persona, Center for Mental Health Care, Nijmegen, The Netherlands; 3 Trimbos Institute (Netherlands Institute of Mental Health and Addiction), Utrecht, The Netherlands; 4 Department of Clinical, Neuro and Developmental Psychology, VU University, Amsterdam, The Netherlands; 5 Department of Epidemiology and Biostatistics, EMGO+ Institute of Health and Care Research, VU University Medical Center, Amsterdam, The Netherlands

**Keywords:** Attention bias modification (ABM), Major depressive disorder, Randomized controlled trial, Economic evaluation

## Abstract

**Background:**

Despite the range of available, evidence-based treatment options for Major Depressive Disorder (MDD), the rather low response and remission rates suggest that treatment is not optimal, yet. Computerized attention bias modification (ABM) trainings may have the potential to be provided as cost-effective intervention as adjunct to usual care (UC), by speeding up recovery and bringing more patients into remission. Research suggests, that a selective attention for negative information contributes to development and maintenance of depression and that reducing this negative bias might be of therapeutic value. Previous ABM studies in depression, however, have been limited by small sample sizes, lack of long-term follow-up measures or focus on sub-clinical samples. This study aims at evaluating the long-term (cost-) effectiveness of internet-based ABM, as add-on treatment to UC in adult outpatients with MDD, in a specialized mental health care setting.

**Methods/design:**

This study presents a double-blind randomized controlled trial in two parallel groups with follow-ups at 1, 6, and 12 months, combined with an economic evaluation. One hundred twenty six patients, diagnosed with MDD, who are registered for specialized outpatient services at a mental health care organization in the Netherlands, are randomized into either a positive training (towards positive and away from negative stimuli) or a sham training, as control condition (continuous attentional bias assessment). Patients complete eight training sessions (seven at home) during a period of two weeks (four weekly sessions). Primary outcome measures are change in attentional bias (pre- to post-test), mood response to stress (at post-test) and long-term effects on depressive symptoms (up to 1-year follow-up). Secondary outcome measures include rumination, resilience, positive and negative affect, and transfer to other cognitive measures (i.e., attentional bias for verbal stimuli, cognitive control, positive mental imagery), as well as quality of life and costs.

**Discussion:**

This is the first study investigating the long-term effects of ABM in adult outpatients with MDD, alongside an economic evaluation. Next to exploring the mechanism underlying ABM effects, this study provides first insight into the effects of combining ABM and UC and the potential implementation of ABM in clinical practice.

**Trial registration:**

Trialregister.nl, NTR5285. Registered 20 July 2015.

## Background

Major Depressive disorder (MDD) is one of the most prevalent mental disorders. With at least 350 million people suffering from this disorder worldwide, it is considered the leading cause of disability, in terms of total years lost due to disability [1]. Cost-of-illness (COI) studies demonstrate a substantial increase of direct (i.e., medical and non-medical costs) and indirect costs (i.e., due to productivity losses and premature death) caused by depression, leading to a high economic burden for all nations [2].

According to the WHO mhGAP Intervention Guide [3], health care for depression preferably consists of a combination of basic psychosocial support with antidepressant medication or psychotherapy, including cognitive behavioral therapy (CBT), interpersonal psychotherapy (IPT) or problem-solving treatment. Although psychopharmalogical and psychotherapeutic interventions are both considered effective in the treatment of MDD (e.g., [4]), less than half of the patients show response and remission to psychotherapy [5] and the majority of patients fail to show remission after a first treatment with standard antidepressant medication [6, 7]. Moreover, depression is characterized by high recurrence rates [8], with up to 85 % of the recovered patients experiencing a new episode during 15 years follow-up (for a prospective study, see [9]), and a substantial increase in risk of recurrence with each successive episode [10].

These rather low response and remission rates, as well as the highly recurrent nature of the disorder indicate, that the underlying factors maintaining depression and predisposing individuals to repeatedly develop new episodes, are not very well understood, yet. In the past years, depression research has therefore extensively focused on the identification of potential vulnerability factors for this disorder [8, 11]. One such cognitive vulnerability factor is a bias in the attentional processing of emotional information [12, 13]. Cognitive theories of depression [14, 15] postulate that information processing in depression is guided by negative schemata, which results in a selective attention for negative, schema-congruent stimuli in the environment. This so-called negative attentional bias is assumed to contribute to both development and maintenance of the disorder.

Indeed, a substantial amount of studies support that, compared to healthy individuals, the depressed show heightened attention for negative compared to positive or neutral information (for a meta-analysis, see [16]), especially when assessed with the dot-probe task [17]. More specifically, the bias in depression appears to operate at a later stage of information processing, as indicated by longer maintained attention on negative stimuli (for eye-tracking studies, see [18–21]) and a difficulty to disengage attention from negative information [22]. However, not only the processing of negative stimuli is impaired in depression. Next to this negative attentional bias, depressed individuals also lack a positive attentional bias, characterized by longer sustained attention to positive than to neutral or negative stimuli, which is usually found in healthy individuals [20, 21, 23].

Both, the presence of negative as well as the lack of positive attentional biases, have been suggested to play an important role in mood regulation. Whereas a positive attentional bias has been associated with a more adaptive emotion regulation under stress [24] and increased resilience [25], the impaired attentional disengagement from negative information has been linked to the ineffective use of emotion-regulation strategies in response to stress [26, 27]. The latter in turn is supposed to lead to prolonged processing of negative information, such as during rumination and hence, to sustained negative affect. Sanchez and colleagues [22] recently provided support for this assumption by showing that in depressed patients, specifically the delayed disengagement from negative stimuli predicts lower recovery from negative mood in response to a stressor.

Based on above-mentioned findings, researches in this field have started to investigate attention bias modification (ABM) paradigms, to alter attentional bias and to examine its causal effects on symptoms in emotional disorders with, so far, main focus on anxious populations [28]. Up to date only a handful of studies have been conducted in depressed samples. Most of these studies made use of the dot-probe task [17] with increased stimulus presentation times (e.g., [29, 30]) to allow for a more elaborate processing of the materials and hence tapping into the attentional bias in depression, which is operating at a later stage of information processing [31].

In a first study, Wells and Beevers [29] showed that a dot-probe training designed at decreasing a negative attentional bias, reduces depressive symptoms in dysphoric students, from baseline to two weeks follow-up, compared to a placebo ABM training, with the group differences being mediated by a change in attentional bias. In a comparable study, a subclinical sample of adolescents with mild to severe symptoms of depression, completed either eight sessions of active, word based-ABM, placebo ABM or assessment-only [32]. Compared to placebo and no-training control group, depressive symptoms reduced significantly in the active group and this decrease was maintained during three months follow-up. These effects were mediated by a decrease in rumination, which was again mediated by a change in attentional bias. Promising results were also found by Browning and colleagues [33], who provided remitted depressed patients 14 days of dot-probe training (two sessions per day) towards positive and away from negative pictures or words. The researchers report an increase in positive attentional bias after the picture-based (but not after the word-based) ABM, as well as reductions in depressive symptoms and cortisol wakening response up to 4 weeks post-training. No such changes were found in the placebo ABM group.

To the best of our knowledge, so far, only two randomized controlled trials (RCTs) have been conducted in samples of currently, clinically depressed individuals. The first study, by Baert and colleagues [34], made use of a different training paradigm than the dot-probe. By means of a spatial curing task, they trained dysphoric individuals’ and clinically depressed patients’ attention away from negative towards positive words. However, no changes in bias were found and symptom improvements appeared only in the dysphoric group. More encouraging findings come from a just recently conducted RCT, investigating effects of a dot-probe based ABM in currently depressed (MDD) patients [35]. Compared to four weeks placebo ABM, the same amount of active ABM successfully reduced a negative attentional bias and increased resting-state connectivity within a neural circuit associated with attentional control over emotional information. Although symptom reduction did not differ across groups, only in the active training group, change in negative attentional bias was associated with symptom improvement, supporting the notion that negative attentional bias maintains the disorder. The decrease of symptoms in the placebo group by contrast, seemed to be related to improved control over spatial attention.

Despite these somewhat mixed results and limited number of studies, above-mentioned research suggests, that ABM might be of therapeutic value for depression and may have the potential to increase response and remission rates, achieved by usual care (UC). Considering the growing acknowledgement of the importance of self-help interventions as adjunct to regular health care in depression [36] and promising findings from numerous studies on the effectiveness of computer-therapy for this disorder (for a meta-analysis, see [37, 38]), the idea would be very appealing to add ABM as internet-based intervention to existing treatment services. As ABM consists of a rather simple and straightforward computer task and thus needs no or minimal assistance of a clinician, it may flexibly be incorporated into patients’ everyday life, being accessible 24 h a day, on every day of the week. Moreover, the training can be followed at home, in a familiar environment and is not associated with any travel time and costs. Due to these advantages, ABM may be provided to patients not only parallel to UC, but would also be accessible during waiting period for treatment. ABM thus might offer patients and effective, acceptable and budgetary affordable intervention to start with immediately after being referred to mental health care and bridge the, due to insufficient treatment capacitates in the health care system, often unacceptable long time between referral and start of treatment (i.e., up to 90 days or more;[39]). Next to the potential therapeutic effects, the addition of ABM on top of regular treatment may have favorable economic impacts through reducing the number of sessions of outpatient care by accelerating speed of recovery. This in turn may reduce down-stream costs and may further positively affect return-to-work and productivity.

Before introducing ABM in treatment programs of depression though, more RCTs are required which address limitations of previous studies. First and most importantly, research needs to investigate the effectiveness of a dot-probe based ABM training in clinical practice. Although at least one RCT has been conducted in a sample of individuals diagnosed with clinical depression [35], the potential of implementing dot-probe based ABM in a specialized clinical health care (and home) setting has not been tested, yet. Second, the long-term effectiveness of ABM still needs to be investigated. Research suggests, that ABM effects may only become apparent over time, when the newly acquainted cognitive processing style is repeatedly deployed in emotional daily-life situations [17, 29, 40]. The previously conducted ABM studies in depressed samples, however, contained either no follow-up [34], or follow-up measures between 2 and 4 weeks [29, 33, 35]. Only one study included follow-up measures at 7 months [32]. If the goal is, to ultimately apply ABM in clinical settings, it is of importance to examine symptom changes across a much longer period of time, in order to see how stable the changes are and whether ABM is associated with higher response and remission rates and, ideally a reduction in relapse percentages. Third, samples sizes of previous studies are rather small, varying between 14 and 29 participants per group [29, 30, 32–34]. Given the supposedly rather small effects of ABM [28, 41], it has been claimed that ABM research should strive for larger sample sizes, to increase the confidence regarding effect size estimates [28, 35, 41].

### Aim and hypotheses

The present study aims to address above-mentioned limitations by means of an adequately powered, randomized, double-blind, placebo-controlled trial, alongside an economic evaluation, investigating the long-term effectiveness and cost-effectiveness of a dot-probe based ABM training, as self-help intervention for clinical depression in a specialized health care setting. One hundred twenty six patients who are diagnosed with MDD and are registered for specialized ambulatory treatment at the mental health care institute Pro Persona in the Netherlands, are randomized into either a positivity training (PT) group (ABM towards positive and away from negative stimuli) or a sham (i.e., placebo) training (ST) group, as control condition (continuous attentional bias assessment). Patients complete eight training sessions (of which seven at home via internet) during a period of two weeks. Changes in attentional bias are assessed from pre- to post-assessment, whereas effects on clinical symptoms are additionally assessed one, six and 12 months after training.

In line with previous research on ABM in depression, we predict that the PT group shows a stronger decrease in negative attentional bias and therefore a stronger decrease in depressive symptoms over time, than the ST group. As already suggested by findings of Wells and Beevers [29] and Beevers and colleagues [35], training effects on depressive symptoms are thus expected to be mediated by an decrease in negative attentional bias. Recent research by Yang and colleagues [32] however suggests, that the ABM effect on depressive symptoms is not directly mediated by change in bias. Instead, a change in bias appears to mediate a change in rumination, which in turn directly mediates the reduction of symptoms. Therefore, we additionally explore the effects of ABM on rumination and it’s mediating role in the effects of ABM on depression.

Although previous research suggests that a negative attentional bias is associated with the use of less effective emotion regulation strategies [26, 42] and hence lower mood recovery from stress [22], research has not tested the causal effects of modifying attentional bias on stress responses in depressed samples, yet. Whereas ABM effects on symptoms are assumed to manifest themselves over a longer period of time (see for instance, [23, 33]), effects on mood reactivity and recovery from stress should be visible shortly after the training already. Hence, the present study also includes a stressful speech task at post-assessment, as a more sensitive measure of the potential therapeutic effects. In line with findings of Sanchez and colleagues [22], it is hypothesized that the PT group shows a higher mood recovery from the stressor than the ST group. Moreover, effects on general levels of resilience over time are measured.

In addition to the effects on depressive symptomatology, rumination and stress responses, this study further extends previous findings by investigating transference effects to other cognitive processes, including attentional bias for verbal emotional information and quality of positive mental imagery, known to be impaired in depression [43]. Furthermore, a measure of cognitive control is included, as previous research suggests that depression is also associated with a lack of inhibitory control over negative information [27] and that ABM can increase activity in neural networks associated with attentional control [35]. Importantly, some previous studies found that sham-training control conditions may lead to comparable changes in symptoms as the active ABM condition, possibly due to the above-mentioned increase in cognitive control, resulting from the contingency-based learning procedure [30, 44–46]. These effects have mainly been observed in socially anxious populations, whereas most studies in depressed samples found active ABM to be more effective than sham ABM [29, 32–34]. Nevertheless, we have to consider the possibility that both groups may show indistinguishable, significant clinical improvements. If this is the case, it will be important to investigate the role of (changes in) cognitive control in the therapeutic effects of the training. At the same time, previous research also suggests that ABM training effects may depend on levels of attentional control (i.e., higher attentional control is associated with stronger training effects; [47]). Therefore, our measure of cognitive control will also be used to explore whether it predicts our training effects on bias.

Finally, recent research strongly recommends adding questions regarding patients’ expectancies, to control for non-specific treatment effects of an intervention [48]. It has been argued that, although active/sham control conditions are superior to waiting-list control groups in controlling for placebo effects, it is only possible to ascribe treatment effects to the intervention, if it can be proven that both, treatment and control group have the same expectations regarding improvement. To our knowledge, this is the first ABM study, which actively controls for possible placebo effects of an ABM training, by measuring participants expectations and the experienced credibility of the training.

## Methods

### Design

**Fig. 1:**
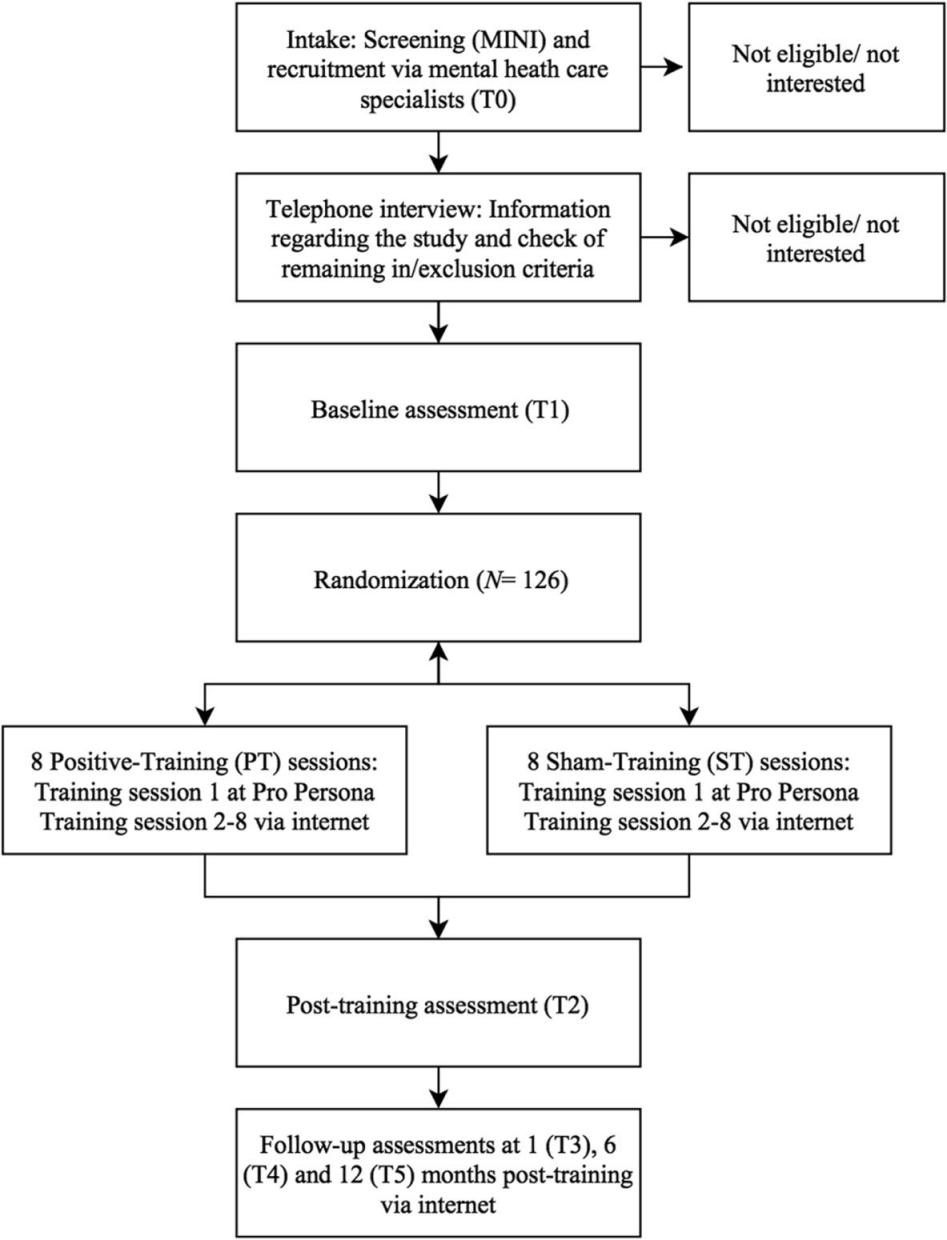
Flowchart of the recruitment and study procedure. *Note*. MINI = Mini International Neuropsychiatric Interview; Measures at T1, Training 1 and T2 are administered at Pro Persona. Training 2–8 and all follow-up measures (T3–T5) are administered via the internet

The study is conducted as a two-arm, double-blinded RCT, at four locations of the Dutch mental health care institute, Pro Persona (Arnhem, Nijmegen, Ede and Tiel). Adult MDD patients are randomly allocated to either a positive ABM training (PT), or a sham ABM training (ST) as control condition. Participants complete a total of eight training sessions distributed over two weeks (i.e., four weekly sessions). The very first session is completed at the mental health care institute and all other sessions via internet, at participants’ home. The training sessions are preceded and followed by an assessment (i.e., a pre- and post-assessment) at Pro Persona and three more follow-up assessments via the internet, one, six, and 12 months later (see Fig. 1 for an overview of the design). The study has received ethical approval by the medical ethics committee Arnhem-Nijmegen (2013/373; NL45720.091.13) and is registered with trialregister.nl, number NTR5285. Any substantial protocol amendments will be communicated to all relevant parties (i.e., medical ethics committee, trial register, journals).

### Participants

#### Inclusion and exclusion criteria

In order to be eligible for participation, Dutch patients between 18 and 65, should meet the criteria for a diagnosis of MDD, first or recurrent according to the DSM- IV-TR [49]^1^ as assessed with the Mini International Neuropsychiatric Interview (MINI; [50]).

Exclusion criteria are (a) any psychotic disorder (current or previous) (b) current mania, hypomania or a history of bipolar disorder (c) acute suicidal risk (d) a verbal IQ < 80 (e) visual disabilities that interfere with a computer task (f) insufficient command of the Dutch language, (g) no regular access to a computer with internet at home, and (h) insufficient experience with the use of computers (based on the subjective estimation of the patient). To maximize recruitment, the use of medication is routinely recorded but does not represent an exclusion criterion. Likewise, the stabilization of the dosage of medication is not required for participation.

#### Recruitment

We recruit 126 outpatients with a major depressive episode (first or recurrent) who are referred to specialty care for ambulatory treatment at Pro Persona. UC in the study is the standard care delivered in the program for mood disorders at Pro Persona according to the Dutch multidisciplinary guideline for depression [51] and can be psychotherapy (e.g., CBT or IPT), antidepressant medication, the combination of psychotherapy and antidepressants or ambulatory care provided by a specialized psychiatric nurse. Health care consumption is closely monitored in both training groups. The MINI is administered as part of the standard intake procedure. Patients who are eligible based on the MINI diagnosis, are informed about the study and the possibility to participate by the mental health care specialists. Those who provide written consent for being contacted by a researcher, are provided more detailed information regarding the study via telephone. During this phone call, remaining exclusion criteria are checked and eligible patients are invited for the baseline session.

#### Randomization and blinding

Participants are randomly allocated to one of the two groups, right before the first training session. Randomization is computerized and stratified for depression severity (<39 = moderate; 39–48 = severe; > 48 = very severe) at baseline, as assessed by the Inventory of Depressive Symptomatology self-report (IDS-SR; [52]), as well as for age (18–35 years; 36–50 years; 51–65 years). In case participants indicate, that they want to discontinue with the study and withdraw consent for using their data, intervention allocation is corrected for the excluded participants.

Based on the IDS-SR score and age, the computer generates a random 5-digit number, which is linked to one of the two training conditions and which further identifies participants and their documents. This blinding code is managed by an independent statistician and revealed to researchers only, after all participants have completed the last assessment. Hence, both participants and researchers are blind to the training conditions.

To assess if participants remained blinded to their group allocation, all participants fill in an awareness check at post-test, investigating which training condition (PT or ST) they believe they have been enrolled to.

## Instruments and materials

### Intervention

#### Attention bias modification

The attention training in this study is based on a modified version of the dot-probe task [17] and is comparable to the task used in the studies of Wells & Beevers [29] and Beevers et al. [35]. For this task, a set of 50 picture pairs was created. Thirty picture pairs (12 × 8 cm) were selected from the Nencki Affective Picture System (NAPS; [53]), with half of the pictures displaying positive scenes or images (e.g., landscapes or puppies) and the other half displaying negative (i.e., dysphoric) scenes or images (e.g., people in sad situations). All picture pairs are matched on content and complexity and are of comparable emotional intensity. Extremely arousing pictures were excluded. Another set of 20 picture pairs (8 × 12 cm) contains exclusively faces from actors (10 male, 10 female), selected from the Umeå University Database of Facial Expressions [54], each of them displaying a happy and a sad expression. An additional set of five neutral NAPS pairs and five neutral Umeå pairs were selected for practice trials preceding the ABM assessment and training.

During each trial of the task, a white fixation cross appears in the middle of the black computer-screen. After 1500 ms, the cross disappears and two pictures, always one positive picture and one negative picture, appear next to each other on the screen – approximately 16 cm apart when measured from the center of each picture. The location of negative and positive pictures (i.e., right or left half of the screen) is counterbalanced across trials. In line with the reference studies by Wells and Beevers [29] and Beevers et al. [30], a left-right oriented stimulus presentation is chosen instead of a top-down orientation. A recent meta-analysis suggests relatively larger effect sizes for left-right than for top-down oriented materials in the dot-probe task [45]. Moreover, the horizontal stimulus orientation has the advantage that on relatively small computer-screens which might be used by participants in this study, the stimuli can be presented in a larger size.

The two pictures are randomly presented for an interval of 1000 or 1500 ms. Following offset of the pairs, a small arrow (i.e., probe) appears in the location of one of the pictures, pointing either up or down, with equal probability. Participants need to indicate the direction of the arrow as quickly as possible, by pressing a corresponding response button on the keyboard. The arrow remains on the screen until the participant reacts to it. The computer records the participants’ reaction time (RT; appearance to disappearance of the arrow) and response accuracy. In case participants do not respond within 3000 ms, they are reminded to do so by the computer.

The whole task contains two sorts of trials: Assessment and training trials. In the 60 assessment trials, a set of 30 out of the 50 picture pairs is presented twice (i.e., 18 NAPS pairs, 12 UMEA pairs). The location of the arrow is counterbalanced across picture valence, such that the arrow replaces negative and positive pictures equally often. During the training trials, all 50 picture-pairs are used and are presented four times each, evenly distributed across four blocks, which are separated by a short break. In the ST, participants receive a continued assessment, during which the probe appears with equal probability in the location of positive and negative pictures. In the PT, contingencies are changed: The arrow appears in about 90 % of the trials in the location of the positive stimulus, such that attention is redirected away from the negative, towards the positive stimuli. A contingency of 90 % is used to allow for assessment of an attentional bias during the training sessions and to keep the intent of the study from being obvious [29]. This contingency is established by evenly distributing 20 additional catch trials across all blocks, during which the arrow appears in the location of the negative picture. In the ST, these extra trials are also added, with the arrow appearing equally often behind the positive and the negative picture. In total, the training part thus contains 220 trials (i.e., four blocks: 50 training trials, five catch trials) and lasts about 20 min.

To increase participants’ motivation and task adherence, feedback is provided during the breaks, including the average response time so far as well as the percentage correct responses. At the end of each training, participants receive feedback on their performance during the whole session, which they are asked to write down in a booklet, such that they can keep track of their performance during the two weeks of training.

Assessment and training trials are preceded by a short block of practice trials. During these trials, five neutral NAPS and five neutral UMEA face pairs are presented, with the arrow appearing with equal probability at each location. Participants repeat the practice trials until they accurately respond to eight of the ten trials. Feedback is provided on each trial: either “correct” when they correctly indicate the direction of the arrow, or “incorrect”, if they incorrectly indicate it’s direction. At post-test, the assessment is followed by 100 re-training (or ST) trials, during which 25 out of the 50 picture pairs are presented four times each (i.e., 15 NAPS pairs, 10 UMEA pairs), to ensure that training effects are not diminished by the post-assessment.

### Primary outcome measures

The main outcome measures are change in attentional bias, change in depressive symptomatology and mood response and recovery from stress.

#### Attentional bias

Change in attentional bias is assessed by means of the dot-probe task. In accordance with previous research [33], an attentional bias score is calculated for each participant, separately for pre- and post-assessment, by subtracting RTs on trials where the arrow replaces the positive picture (compatible trials) from trials where the arrow replaces the negative picture (incompatible trials). A positive score indicates an attentional bias towards positive pictures, whereas a negative score indicates a bias towards negative pictures.

#### Depressive symptoms

Changes in depressive symptoms are assessed with the IDS-SR self-report [52]. The IDS-SR contains 30 items that are rated on a 4-point scale (0 to 3). Possible total scores can thus range from 0 to 90 with higher scores indicating greater depression severity (i.e., 0–13 = *no depression*; 14–25 = *mild depression*; 26-38 = *moderate depression*; 39–48 = *severe depression*; > 48 = *very severe depression*. The IDS-SR is well validated and has been proven useful in detecting symptom change and residual symptoms in depressed patients [55].

#### Mood reactivity and recovery from stress

To investigate training effects on mood changes in response to a stressor, an adapted version of a speech task as used by Sanchez et al. [22] is used. At the beginning of this task, participants sit in front of a black computer screen and rest for a period of five minutes. Afterwards, they rate their mood, and are then informed by means of computer instructions, that they will get two minutes to prepare a five-minute-speech on the topic – e.g., “Why am I a good friend?”. This topic has effectively induced stress levels in patients with MDD in the study of Sanchez and colleagues. Participants are told that the speech will be video recorded and evaluated on its quality by two independent researchers. However, they are also informed that not everybody would have to give the speech eventually. Instead, the computer would randomly choose, who actually has to give the speech. During the following two-minutes of preparation, participants are allowed to take notes with pen and paper and, to increase the stress level, a clock on the computer-screen signals the time left. After preparation, participants again rate their current mood before the computer informs them, that they would not have to give the speech. They are then given another five minutes to rest and rate their mood for a last time afterwards. This procedure has successfully been used by Sanchez and colleagues to investigate the relation between attention bias and stress responses in depressed individuals.

Mood changes during the speech task are measured by means of a six-item, state version of the Spielberger State-Trait Anxiety Inventory (STAI; [56, 57]) and a state version of the international short form of the Positive and Negative Affect Schedule (I-PANAS-SF; [58]). The STAI-S contains six items related to anxiety (i.e., calm, tense, upset, relaxed, content, worried) that are rated on a 4-point scale (1 = *not at all* to 4 = *very much*). This short form of the STAI has been proven sensitive to fluctuations in state anxiety and shows comparable psychometric properties as the full-form [56]. The I PANAS-SF contains five items related to positive and five items related to negative affect, rated on a five-point scale (1 = *very slightly* or *not at all* to 5 = *extremely*) and has shown to be psychometrically acceptable [58].

### Secondary outcome measures

#### Trait anxiety

In line with earlier research on the effects of repeated Cognitive Bias Modification (CBM) sessions in depression [33], the 20-item Spielberger State-Trait Anxiety Inventory (STAI-T; [59]), is used to assess anxiety proneness. All items are rated on a Likert-scale ranging from 1 (*not at all*) to 4 (*very much*). The Dutch translation of the scale exhibits good psychometric properties [60, 61].

#### Positive and negative affect

To assess changes in general mood, the Positive and Negative Affect Schedule (PANAS; [62, 63] is used. The PANAS is a 20-item self-report measure consisting of two mood scales, one assessing positive and one assessing negative affect. Items are rated on a 5-point scale ranging from 1 (*very slightly or not at all*) to 5 (*extremely*). The Dutch translation of the PANAS has adequate psychometric properties [64].

#### Rumination

Ruminative thinking is assessed with the Ruminative Response Scale (RRS-NL), a subscale of the Response Styles Questionnaire (RSQ; [65]). This self-report scale contains 22 items assessing an individuals’ tendency to engage in rumination that is either self-focused (e.g., “think what am I doing to deserve this?”), symptom focused (e.g., “think about how hard it is to concentrate”) or focused on causes and consequences of having a depressed mood (e.g., “think I won’t be able to concentrate if I keep feeling this way”). Items are rated on a 4-point scale, ranging from 0 (*almost never*) to 4 (*almost always*). Next to a total rumination score, this questionnaire provides brooding and reflective pondering subscale scores, with brooding being considered the maladaptive component of rumination, which is associated with more depression [66]. The psychometric properties of the Dutch translation of the scale have been proven to be moderate to good [67].

#### Resilience

Resilience is assessed with the Resilience Scale (RS; [68]). This self-report questionnaire contains 25 items (e.g., “I feel that I can handle many things at a time.“), rated on the four-point scale (i.e., 1 = *strongly disagree* to 4 = *strongly agree*). The Dutch translation of the scale has been shown to be a reliable and valid instrument to measure the degree of individual resilience [69].

#### Cognitive transfer measures

To assess transfer to an attentional bias for verbal negative and positive information, the *emotional Stroop task* [70] is used. This task consists of three randomized blocks with valenced words (negative, neutral, and positive) that are presented in red, yellow, green, or blue on a computer screen. The words are selected from a database [71] and are matched for length and valence strength. Participants are instructed to indicate the ink color by naming the color of the word aloud, while ignoring the meaning of the words. The experimenter records reaction time (RT) and errors per block. A bias score is calculated by subtracting the RTs for the positive (negative) block from the reaction time for the neutral block. A positive score is indicative for an attentional bias for positive (negative) words.

The classical *Stroop color naming task* [72] is administered in order to (1) explore whether trait differences in cognitive control predict training effects on bias, and (2) to assess transfer effects of the training on cognitive control. As in the emotional Stroop task, participants have to name the color of words or crosses while ignoring their content. On neutral trials, rows of colored crosses are presented (e.g., “xxxx” printed in green). On congruent trials, color words are presented while the meaning of the word and its print color match (e.g., the word “green” printed in green). On incongruent trials, the meaning of the color words and its print color do not match (e.g., the word “green” printed in red). On these trials, participants thus have to inhibit their automatic response to read the word, resulting in slower RTs and more errors when naming the colors compared to on neutral trials. RTs to neutral (congruent) trials are subtracted from RTs to incongruent trials. Higher positive scores accordingly are indicative for less cognitive control.

In order to assess whether effects of the PT generalize to other forms of cognitive biases in depression, in this case the reduced ability to generate positive mental imagery of future events, the Prospective Imagery Task (PIT; [73, 74]) is used. Participants have to form mental images of ten positive and ten negative future scenarios (e.g., “You will have lots of energy and enthusiasm” or “You will feel misunderstood”) and to rate the vividness of each image on a 5-point scale ranging from 1 (*not imagine at all*) to 5 (*very vivid*). To obtain further information on the quality of the generated images, participants rate how much they feel as though they are actually experiencing the event while imagining it (1 = *not at all*; 5 = *completely*), how likely they think it is that the scenario happens to them in the future (1 = *not at all likely to occur*; 5 = *extremely likely to occur*) and how much they feel as though they are actually experiencing the event while imagining it (1 = *not at all*; 5 = *completely*). Although, for this study, we are primarily interested in positive mental imagery, we also include the negative items to allow for control of the general ability to generate prospective mental imagery. Internal consistency has been calculated for a Dutch sample by Blackwell and colleagues [75], where all subscales demonstrated good internal consistency (*α =* 0.83–0.90). At the end of the task, participants are asked to estimate how often they had retrieved a memory from a past event in order to vividly imagine the described scenario (i.e., 0–100 % of the cases). To control for everyday use of mental imagery when analyzing effects on positive imagery ability, the Spontaneous Use of Imagery Scale (SUIS) [76] is administered. The scale consists of 12 items, such as “If I am looking for new furniture in a store, I always visualize what the furniture would look like in particular places in my home”, that are rated on a 5-point scale (1 = *never appropriate*; 5 = *always appropriate*). The SUIS has good psychometric properties and Blackwell et al. [75] report good internal consistency in a Dutch sample (*α* = 0.81).

#### Process measures

Training task performance is assessed for both the PT group and the ST group by comparing average RTs for incompatible trials (in the PT: catch trials) to average RTs for compatible trials across training blocks and sessions. Based on this, learning curves within each session and losses of training effects between sessions will be investigated. To evaluate effects of the online training sessions on mood, three visual analogues scales (VAS) are administered prior to and following each dot-probe session. The VAS scales were adopted from Sanchez et al. [22] and consist of three items each: happy mood (happy, optimistic, joyful), anxious mood (nervous, tense, anxious), and sad mood (depressed, upset, sad). Participants are asked to indicate their current mood on a line with anchor points ranging from 0 (*not at all*) to 10 (*very much*).

To control for possible placebo effects of the intervention, the Credibility/Expectancy Questionnaire (CEQ; [77]) is used. The CEQ is a short self-report questionnaire that measures how credible and convincing the patient experiences the intervention and what the patient expects from it. The questionnaire in this study has been adapted from a Dutch version of this questionnaire for chronic back pain patients [78]. It comprises three questions regarding the credibility of the intervention and three questions assessing participants expectations, all of which are answered on a 9-point scale (1 = *not at all* to 9 = *very much*).

#### Cost-effectiveness

The Trimbos/iMTA questionnaire for Cost associated with Psychiatric Illness (TiC-P; [79]) is used to measure direct medical, direct non-medical and indirect non-medical costs resulting from health care uptake and productivity losses associated with MDD. The TiC-P is the most widely used health service receipt interview for economic evaluations in the Netherlands. The EuroQol (EQ-5D-3 L) is administered to measure health related quality of life [80]. It is a frequently used, preference-based, generic index instrument for the calculation of health-related quality of life in terms of quality adjusted life years (QALYs; [81]).

### Procedure

Eligible patients who agreed to participate, receive a printed version of the TiC-P and are asked to fill it in at home and bring it to the baseline session (session 1). At start of this session, participants again receive written and oral information about the study. Then, they provide written informed consent and hand in the filled-in TiC-P. In a computer room, first of all, a range of disorder-related characteristics are assessed by the experimenter, including the age of onset of the depression, the number of depressive episodes during life time, the amount of psychological treatment for previous episodes, as well as information about the current episode (i.e., duration, number of psychological treatment sessions, and duration and dosage of pharmacological treatment). Afterwards, participants are seated in front of a computer, where they complete the dot-probe task (pre-assessment), which is preceded by a brief assessment of mood state (VAS). Next, they fill in computerized versions of all questionnaires (i.e., IDS-SR, STAI-T, RRS, RS, PANAS, SUIS, EQ-5D-3 L), before they complete the Stroop tasks. Finally, the PIT is administered and an appointment is made for the first training session, which again takes place at Pro Persona, within 5 days after the baseline session. Although the training is offered as web-based intervention, participants complete the very first training session under supervision of a researcher, to ensure that task instructions are well-enough understood and that the task can be completed correctly at home. The importance of completing the training by themselves, in a calm, distraction-free environment (i.e., without any music, television etc. on the background) is emphasized, both verbally and in the written task instructions. This is of particular relevance, as previous research suggests that when working memory load is high, which is likely the case in a home-environment with task-irrelevant distractions, ABM may be less or not effective [82].

During the first training session (session 2), participants receive written and verbal practical information regarding the online training-program. They then complete the first training which is, like all dot-probe trainings, flanked by brief mood state measures (VAS). Afterwards, the CEQ is administered and appointments are made for the seven training sessions (session 3–9), as well as for the post-assessment (session 10). The eight training sessions are distributed across two weeks, with four weekly sessions. During the two weeks of training, participants receive automated e-mails reminding them of the planned sessions. In case, participants do not complete the planned sessions, even though they have been reminded by e-mails, they are given a call by the experimenter who asks them to still complete the session. The post-assessment, which again takes place at Pro Persona, is scheduled at least one week and a maximum of two weeks after the last training session. The post-assessment is comparable to the baseline assessment but without the Tic-P and the RS, and with the additional speech task. Afterwards, appointments are made for the subsequent follow-up assessments and participants receive a reimbursement for their travel cost (15 Euros).

During the first two follow-up assessments (session 11 & 12), participants complete questionnaires via the internet (IDS-SR, STAI-T, RRS, RS, PANAS, EQ-5D-3 L). At 12 months follow-up (session 13), the MINI is administered via telephone to assess the diagnostic status of depression. Moreover, participants complete the IDS-SR, EQ-5D and Tic-P via internet. After having filled in the questionnaires, participants receive a debriefing form with contact information of the coordinating investigator, who can be contacted in case of questions. The use of medication as well as the number of psychological treatment sessions is monitored at the post-test and all follow-up measures. For an overview of all measures and corresponding time points, see Table 1.

**Table 1:** Overview of all measures and corresponding time points

Domain	Target Concept (Measure)	T0	T1	Training 1	Training 2–8	T2	T3	T4	T5
Screening measures	Inclusion Criteria Interview	•							
	Diagnostic status (MINI)	•							•
Primary outcome measures	Attentional Bias (Dot-probe)		•			•			
	Depressive Symptomatology (IDS-SR)		•			•	•	•	•
	Mood response and recovery from speech task (I PANAS-SF & short STAI-S)					•			
Secondary outcome measures	Trait Anxiety (STAI-T)		•			•	•	•	
	Positive and Negative Affect (PANAS)		•			•	•	•	
	Rumination (RRS)		•			•	•	•	
	Resilience (RS)		•				•	•	
	Cognitive transfer -Verbal attentional bias (emotional Stroop task) -Attentional control (classical Stroop task) -Mental imagery (PIT, SUIS)		•			•			
Process measures	Attentional Bias (Dot-probe training trials)			•	•				
	Mood in response to training (VAS)			•	•				
	Credibility and Expectancy (CEQ)			•					
Cost-effectiveness	Quality of Life (EQ-5D-3 L)		•			•	•	•	•
	Costs (TIC-P)		•						•

*Note*. T0 = Intake; T1 = Baseline; T2 = Post-assessment; T3 = One month follow-up; T4 =Six months follow-up; T5 = 12 months follow-up; Measures at T1, Training 1 and T2 are administered by an experimenter, who is blinded to the training conditions. Training 2–8 and all follow-up measures (T3–T5) are administered via the internet

### Data-handling

The data is securely stored in a folder with limited access by the involved researchers only and will be retained for 15 years after inclusion. To assure confidentiality, participant data (both paper-based and electronic data) is identified by a participant ID (see randomization and blinding). Electronic data is protected by a password. The file linking participant ID and personal information is stored in a separated file which is also password protected. To ensure data-quality, paper-based data entry will be double checked. Only the principal investigator and first author will have access to the final data-set. As the study involves low to negligible risk, no additional data monitoring committee is assigned. Results of this study will be published in national and international journals and will be presented at conferences. Moreover, participants and involved clinicians will receive a summary of the study results.

### Analyses

#### Sample size

Wells and Beevers [29] reported a large effect of ABM on change in bias (*η* ^*2*^ = .16) and on depressive symptoms (*η* ^*2*^ = .32). Likewise, Beevers et al. [35] observed a large effect for change in bias from pre- to post-ABM within the training condition (*d* = 1.01, *η* ^*2*^ = .20), but not the control condition (*d* = .04, *η* ^*2*^ = .0004). However, the above-mentioned studies were conducted in a sample of dysphoric students [29] and in a sample of treatment-seeking current MDD patients, some of whom received pharmacological treatment [35]. In the present study, ABM is provided during the waiting period for psychotherapy or in combination with pharmacological treatment and/or psychotherapy (i.e., IPT, CBT, psychiatric care). In order to account for the greater heterogeneity of our sample and because recent meta-analyses of the effects of CBM in anxiety and depression suggest rather small effects [28, 41], we conservatively assume a small to medium effect size of *η* ^*2*^ = .02 (i.e., *f* = .142). A-priori sample size is calculated with G*Power for F-tests for repeated-measures (RM) Analysis of Variance (ANOVA) with within-between interaction effects and an effect size specification as recommended by Cohen [83]. To be able to detect this effect size over two time-points, with an α-level of .05 and a power-level of .80, a total sample size of 100 would be needed, thus 50 participants per group. Compensating for an expected attrition of 20 %, we need to include 50/0.80 = 62.5, thus 63 participants per condition and *N* = 126 in total.

### Analysis plan

#### Clinical evaluation

In accordance with the Consolidated Standards of Reporting Trials (CONSORT; [84]), all analyses of the primary and secondary outcome parameters will be carried out in accordance with the intention-to-treat (ITT) principle. Missing values due to drop-out will be imputed (e.g., by using multiple imputation (MI) or expectation maximization (EM)). Additional sensitivity analyses will be conducted to gauge the robustness of the findings, by including and excluding these participants in the analysis.

ABM effects on the primary outcome measures, attentional bias and depressive symptoms, will be tested by means of RM ANOVAs, with condition (PT, ST) as between-subjects factor and time (pre-test, post-test) as within-subjects factor. Likewise, training effects on stress responses will be investigated with a 2 (condition: PT, ST) × 3 (time: before stress, anticipatory stress, stress recovery) RM ANOVA with I PANAS-SF and STAI-S scores as dependent variables. All analyses will be conducted with and without covariates, in case variables are unevenly distributed across groups (e.g., bias or depression at baseline, number of previous depressive episodes, or number of completed training sessions), to test the robustness of the results. To investigate long-term effects of ABM on depressive symptoms, the analysis of this primary outcome parameter will be repeated over five time points (baseline, post-test, one, six, and 12 months follow-up).

Effects on secondary outcome measures will be analyzed by means of ANOVAs or ANCOVAs over two time points (baseline, post-test) in case of the cognitive transfer measures, and over four time points (baseline, post-test, one and six months follow-up), in case of rumination, positive and negative affect, and resilience.

Exploratory analyses will be directed at the process measures and will additionally be performed to take the role of potential moderators into account, such as timing of the training (i.e., during waiting period vs. next to UC) type of UC (IPT, CBT, psychiatric care), or to predict training effects based on other disorder-related characteristics assessed during baseline.

Finally, mediation analysis will be conducted to investigate whether change in attentional bias across sessions mediates the relationship between training condition and depressive symptoms. A second exploratory mediation analysis will test the mediating effect of rumination. This analysis will only include participants who completed an adequate dose of training sessions (i.e., 6–8).

As alternative to general linear models (GLM), which have been the standard approach to analyzing data of similar trials as the one proposed, more recent statistical developments including linear mixed models (LMM) might be considered when analyzing the data.

#### Economic evaluation

The Economic evaluation will be conducted in two ways. First, we will conduct a cost-effectiveness analysis (CEA) with treatment (i.e., UC + PT versus UC + ST) response as the clinical outcome of interest. A probabilistic medical decision-making approach is chosen, as costs are associated with large standard deviations and powering the trial for testing an economic hypothesis would require an extremely large sample size. Treatment response will be based on a binary outcome, namely the clinical outcome “depressive symptoms” (i.e., the IDS-SR score), and will be computed as a 50 % (or better) pre/12 months follow-up symptom IDS-SR reduction. Second, we will conduct a cost-utility analysis (CUA) using quality adjusted life years (QALYs, based on the EQ-5D-3 L) as a generic measure of health gains. Both the CEA and the CUA will be conducted from the societal perspective and in agreement with the ITT principle. The trial’s follow-up measurements will not exceed one year and therefore neither costs nor effects will be discounted. Sensitivity analyses will be directed at uncertainty in the main cost-drivers.

Four types of costs will be considered: (1) the costs of offering the intervention, (2) costs stemming from health care uptake including the costs of medication, (3) patients’ out-of-pocket costs, (4) costs stemming from productivity losses due to absenteeism and lesser efficiency while at work. The first two types of costs are also known as the direct medical costs and these will be based on the full economic costs of offering the training. For this we shall make use of the pertinent Dutch guideline for economic evaluation [85] and use the standard cost prices reported therein for the reference year 2014. The patients’ out-of-pocket costs are also known as direct non-medical costs and encompass the patient’s costs of travelling to health services, parking costs of the patient while travelling to and from treatment. Finally, productivity losses will be based on the mean friction costs, as outlined in the Dutch guideline for costing. Data on resource use (health care uptake) and productivity losses will be collected with the latest version of the TiC-P [79]. Importantly, in both conditions, costs of health care uptake (UC) will be evaluated. However, only in the PT condition, the costs of the intervention will be added, because that would best reflect the incremental costs of adding the new intervention onto the current package of UC and thus helps to answer the relevant question, if adding the new interventions offers good value for money.

The incremental cost-effectiveness ratio (ICER) will be computed to obtain the costs per treatment response and the costs per QALY gained. Stochastic uncertainty will be handled using 2500 non-parametric bootstraps and plotting the simulated ICERs on the ICER plane. For decision-making purposes, the ICER acceptability curve will be plotted for various willingness-to-pay (WTP) ceilings, which helps to making judgments whether the intervention offers good value for money relative to UC without PT. One-way sensitivity analyses directed at uncertainty in the main cost drivers will be performed to gauge the robustness of our findings across a range of likely values of those parameters.

## Discussion

Despite the range of available, evidence-based treatment options for MDD, the rather low response and remission rates [5–7] suggest that treatment for depression is not optimal, yet. Considering the increasing demand for mental health care, the associated costs and limited resources, adding easily accessible computerized interventions as adjunct to regular treatment services of depression, is an appealing and likely efficient option when aiming at treatment optimization.

ABM might have the potential to be provided as such a cost-effective intervention during waiting period for UC or next to the regular treatment sessions by bringing more patients into remission and by reducing face-to-face sessions, especially when being provided via internet. Although the number of ABM studies in depressed samples is still limited and findings are somewhat mixed [34, 35], existing literature provides encouraging evidence that ABM may indeed decrease depressive symptoms in sub-clinically depressed [29] and clinically depressed individuals [29, 32, 35] and thus may have therapeutic value. Most of these previous studies have however limited their scope, by focusing on ABM effects on depressive symptoms and rumination in non-clinical samples, outside mental health care institutions. Moreover, only one RCT has been conducted so far, testing the long term effects on symptoms (i.e., up to seven month after training [32]).

To the best of our knowledge this is the first RCT examining the long-term effectiveness and cost-effectiveness of an internet-based ABM intervention in clinically depressed patients, in a specialized mental health care setting. Based on the extensive literature showing that depression is characterized by heightened attention for negative information and a lack of attention for positive information [86], we investigate whether a dot-probe based ABM training away from negative and towards positive pictures is therapeutically effective compared to a placebo (i.e., sham ABM) training. Moreover, it is tested whether ABM effects on symptoms are indeed mediated by a change in attentional bias, as suggested by earlier research [29].

Next to the long-term effects on clinical symptoms of depression, the present study further aims at extending findings of previous studies, by investigating whether the training affects emotion regulation in response to stress and general levels of resilience. Regarding the mechanisms of symptom change, it is assumed that effects on symptoms manifest themselves over a longer period of time, after the bias has been repetitively deployed in stressful daily life situations [17, 40]. Although there is evidence that the prolonged processing of negative information is related to impaired mood recovery after stress [22], this is the first study examining the causal link between attentional bias and stress responses in depression.

Moreover a range of exploratory questions are addressed. To get more insight into mechanisms associated with symptom change, we additionally test the mediating role of rumination in ABM effects. Second, transference effects to attentional bias for verbal materials and to other cognitive measures, including cognitive control and prospective positive mental imagery are investigated. Third, the credibility of the training and related expectancies are measured and controlled for. The latter will not only provide insight into the role of placebo effects within ABM research, but may also give an indication of the acceptance of such a computerized training among patients.

Altogether, this study will enhance our understanding regarding the role of attentional bias in depression and the potential therapeutic effectiveness of ABM in this population. To our knowledge, this is the first study testing the cost-effectiveness of an ABM training in clinically depressed patients in a specialized mental health care setting. It thereby also provides insight into the combination of UC and ABM and whether adding such an internet-based intervention, requiring limited to no time of experienced clinicians, is indeed economically sensible. If the training shows the expected beneficial effects, this study will form an important step towards the implementation of ABM in clinical practice and the optimization of UC through computerized self-help trainings.

## Abbreviations

ABM: Attention bias modification
ANOVA: Analysis of variance
APA: American Psychiatric Association
CBM: Cognitive bias modification
CBT: Cognitive behavioural therapy
CEA: Cost-effectiveness analysis
CEQ: Credibility/Expectancy questionnaire
COI: Cost-of-illness
CONSORT: Consolidated Standards of Reporting Trials
CUA: Cost-utility analysis
DSM-IV: Diagnostic and statistical manual of mental disorders 4^th^ edition
EM: Expectation maximization
EQ-5D: EuroQol
GLM: General linear models
HSCIC: Health & Social Care Information Centre
I PANA-SF: Short form of the international positive and negative affect schedule
ICER: Incremental cost-effectiveness ratio
IPT: Interpersonal psychotherapy
ITT: Intention-to-treat
LMM: Linear mixed models
MDD: Major depressive disorder
mhGAP: Mental Health Gap Action Programme
MI: Multiple imputation
MINI: Mini International Neuropsychiatric Interview
NAPS: Nencki affective picture system
PANAS: Positive and negative affect schedule
PIT: Prospective imagery task
PT: Positivity training
QUALY: Quality of adjusted life years
RCT: Randomized controlled trial
RRS-NL: Ruminative response scale
RS: Resilience scale
RSQ: Response styles questionnaire
RT: Reaction time
ST: Sham training
STAI: Spielberger state-trait anxiety inventory
SUIS: Spontaneous use of imagery scale
Tic-P: Trimbos/iMTA questionnaire for cost associated with psychiatric illness
UC: Usual care
VAS: Visual analogue scale
WHO: World Health Organization
WTP: Willingness-To-Pay

## Footnotes

1 The DSM-IV-TR is still used for diagnostic purposes at mental health care institutions in the Netherlands until 2017. Therefore, the inclusion criteria in this study are still based on this former DSM version, instead of on the DSM-V. As the main criterion symptoms applied to the diagnosis of a major depressive episode and the requisite that these symptoms have to be present for at least two weeks do not differ between the two versions of the DSM [87], relying on the DSM-IV-TR for inclusion is not considered problematic.
